# Retinal Imaging of Infants on Spectral Domain Optical Coherence Tomography

**DOI:** 10.1155/2015/782420

**Published:** 2015-07-06

**Authors:** Anand Vinekar, Shwetha Mangalesh, Chaitra Jayadev, Ramiro S. Maldonado, Noel Bauer, Cynthia A. Toth

**Affiliations:** ^1^Narayana Nethralaya Postgraduate Institute of Ophthalmology, Bangalore, India; ^2^Department of Ophthalmology, Duke University, Durham, NC, USA; ^3^Department of Ophthalmology, Maastricht University, Netherlands

## Abstract

Spectral domain coherence tomography (SD OCT) has become an important tool in the management of pediatric retinal diseases. It is a noncontact imaging device that provides detailed assessment of the microanatomy and pathology of the infant retina with a short acquisition time allowing office examination without the requirement of anesthesia. Our understanding of the development and maturation of the infant fovea has been enhanced by SD OCT allowing an in vivo assessment that correlates with histopathology. This has helped us understand the critical correlation of foveal development with visual potential in the first year of life and beyond. In this review, we summarize the recent literature on the clinical applications of SD OCT in studying the pathoanatomy of the infant macula, its ability to detect subclinical features, and its correlation with disease and vision. Retinopathy of prematurity and macular edema have been discussed in detail. The review also summarizes the current status of SD OCT in other infant retinal conditions, imaging the optic nerve, the choroid, and the retinal nerve fibre in infants and children, and suggests future areas of research.

## 1. Introduction

Optical coherence tomography (OCT) is a routine diagnostic imaging method used worldwide in the evaluation of vitreoretinal diseases in the adult population and has become the indispensable cornerstone of posterior segment disease management over the past few decades. Despite its unquestioned utility in adult clinical practice, it has taken much longer to be used, explored, and understood in pediatric retinal imaging.

In particular, spectral domain OCT (SD OCT) imaging of infants is a relatively new field and has opened up new areas of clinical research in our understanding of the anatomy, as well as evaluating the pathology in these eyes. The aim of this review is to summarize some of the most current and relevant literature on SD OCT imaging of infants and to discuss current and future trends in this field.

*Spectral versus Time Domain OCT*. Spectral domain optical coherence tomography (SD OCT) measures the interference spectrum of time delay echoes of light using a spectrometer. This device is based on a high-speed charge coupled device (CCD) camera. Interference spectrum is based on the principle of oscillations and is proportional to the reflected time delay. These scans are produced by a mathematical operation, which extracts the frequency content of this signal. The extracted measure is called the Fourier transformation. The main advantage of SD OCT over the previous technology of time domain OCT or TD OCT is that the former provides better resolution and faster acquisition speeds. This makes it more efficient especially in pediatric imaging, where accurate scans must be obtained in the shortest period of time. Between 25,000 and 100,000 A scans are routinely possible using SD OCT which is >100 times faster than the TD devices allowing image capture even in the uncooperative neonate [1].

## 2. Historical Aspects

Historically, the chief limitation of imaging infants with OCT has been the lack of available equipment designed to allow quick and easy acquisition of images especially in these unanesthetized, uncooperative, preverbal patients. The limited popularity of SD OCT imaging in infants and young children has been because of the dependence on older equipment that did not allow easy image acquisition in the supine infant. Pediatric patients often needed to be anesthetized or sedated, in an operating room with a team comprised of a pediatric nurse and anesthetist. To allow easier examinations, several modifications have been described, such as, the “flying baby” position which allowed the infant to be horizontally held, albeit under monitoring in the operating room. This allowed images to be acquired upright without the problem of lateral inversion [2].

Previously, the tabletop OCT device which is a vertical imaging system has been converted to a hand-held system for supine imaging [3, 4]. This has been described using the Spectralis (Heidelberg Engineering, Germany) and is accomplished in a two-step disassembly which frees the camera, allowing the user to align the axis in any plane for capture [4]. Since the infant is supine and needs no anesthesia, this can be performed in the office as well, although monitoring by an anesthetist is advisable. However it must be remembered that since the position of the infant's head is closer to the camera and the technician stands at the head end of the infant, the resulting images are inverted. This is important while interpreting localization of the lesion under study. Using a full-fledged adult device like the Spectralis allows the added advantage of performing fluorescein or indocyanine green angiography simultaneously while acquiring the OCT images [4].

More recently, with the commercial availability of a hand-held OCT device, Envisu (Bioptigen, NC, USA), imaging of infants and children has become simpler. Using this device, infants can be imaged conveniently in the office without anesthesia [4–9] or in the operating room [10, 11], as the clinical situation demands. The device allows for the reference arm to be switched between the anterior and posterior segment. The focus is adjusted manually using the noncontact camera and can be performed even through an undilated pupil. The manufacturer-supplied calibration factor allows the conversion of the reference arm read-out to an optical distance measured in millimeters and provides a focus correction with a range of +10 to −12 diopter (D).

## 3. Optimizing Image Acquisition

While considering imaging of pediatric eyes on the SD OCT, we must understand that these eyes are unique in refractive growth and, hence, optics is an important consideration for optimized image capture.

Important differences of the infant eye are as follows:

1. The axial length increases rapidly in the neonatal period and grows approximately 0.16 mm per week [12, 13]. This growth slows with age from approximately 1 mm/year during the first 2 years to 0.4 mm/year from 2 to 5 years and to 0.1 mm/year from 5 to 15 years. After age 15, no significant further growth occurs.
2. Similarly, the refractive error (RE) varies with age and differs from an adult. Cook et al. reported an RE of −2.00 D at 32 weeks and −1.23 at 36 weeks, with a shift to hyperopia (+0.74 to +2.12) by 40 to 52 weeks
3. The neonate's cornea is steeper than the adult's cornea, with a mean central corneal power between 48 and 58.5 D, decreasing to adult values by three months [13].
4. Astigmatism is also noted to be greater than in adult eyes and decreases by 50% in approximately 6 months [14].
5. While imaging the retina using adult eye settings, the OCT scanning pivot location is displaced anteriorly relative to the pupil, and the peripheral portion of the image is clipped causing loss of peripheral information. This may be overcome by shortening the OCT reference arm delay such that the pivot point is returned to the pupil plane.

Considering the deviation in optics of an infant eye compared to standard adult eye optics, Maldonado et al. recommended age-specific considerations in the SD OCT imaging protocol for young children, which is summarized in Table 1 [8]. This included changing the reference arm position, focus, and scan settings based on age. For example, in a 32-week postmenstrual age (PMA) infant, each millimeter of presumed scan length would actually be 0.629 mm at the retina (62.9% of the adult eye). Therefore, performing a 10 mm retinal scan (set for an adult eye) would result in a 6.3 mm retinal scan in this infant's eye.

## 4. Understanding Normal Foveal Development through SD OCT

SD OCT has offered us an in vivo imaging tool to study the different layers of the infant fovea. This is based on the intrinsic reflectance property of tissues or the interface between adjacent layers. In the retina, the contrast between alternating layers of lower reflective cell nuclei and higher reflective axons, dendrites, and melanosomes allows easy differentiation [15].

**Figure 1:**
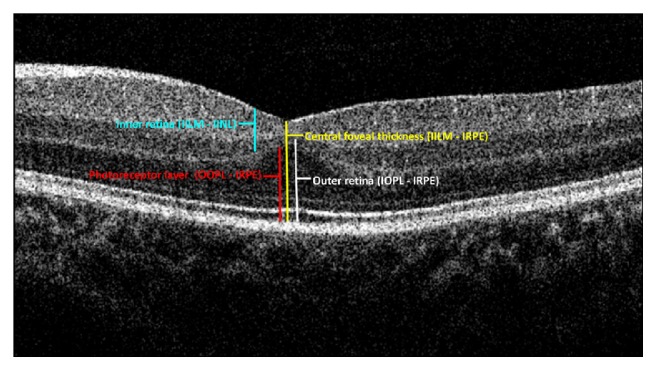
The zones and the layers marked on the SD OCT image of a neonate.

Before we understand the development of the fovea in infants, we must be familiar with some definitions used to describe the layers and zones (Figure 1):

1. Central foveal thickness (CFT, yellow line) which is the thickness of the entire retina from the inner aspect of the inner limiting membrane (ILM) to the inner aspect of the retinal pigment epithelium (RPE) at the foveal center.
2. Inner retinal layers (IRLs, blue line) which includes all retinal tissue from the inner aspect of the ILM to the outer border of the inner nuclear layer (INL).
3. The outer retinal layers (ORLs, white line) which extends from the inner aspect of the outer plexiform layer (OPL) to the inner border of the RPE.
4. The photoreceptor layer (PRL, red line) which extends from the outer aspect of OPL to the inner border of RPE.

Compared to adult foveae, preterm infant foveae differ on SD OCT by demonstrating

1. a visibly shallower foveal depression;
2. presence of persisting inner retinal layers including the inner plexiform and the inner nuclear layers (the so called “inner retinal immaturity”);
3. thinner retinal layers overall;
4. attenuation of the PRL with absence of photoreceptor sublayers.

**Figure 2:**
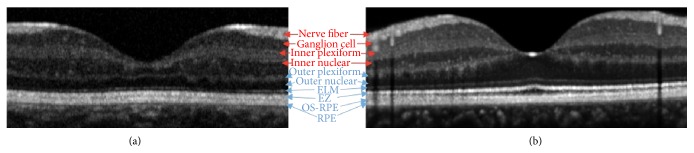
Retinal layers (red are inner retinal layers and blue are outer retinal layers) are compared between PMA 38-week preterm infant (a) and 38-year-old adult (b).

The retinal layers imaged on SD OCT are compared between a 38-week-old premature infant and a 38-year-old adult in Figure 2.

### 4.1. Inner Retinal Development

The persistence of the IRLs in the foveal center is characterized by the presence of the ganglion cell layer (GCL), inner plexiform layer (IPL), and the INL as distinct measurable layers at the foveal center. These condense into a single thin hyperreflective band in children and adults.

The thickness and number of IRLs at the foveal center decrease over time as the premature infant eye matures. This causes deepening of the foveal pit. Most of this thinning occurs by inner retinal cell migration centrifugally and occurs between 31 and 42 weeks of PMA [16, 17]. This further leads to the decrease in the foveal-to-parafoveal inner retinal layer thickness ratio with increasing age. This centrifugal cell migration of the IRL to form the foveal pit has been confirmed on three-dimensional maps. This is consistent with the progressive increase in the height of a parafoveal annulus of the retina. Most of this IRL migration occurs between 31 and 42 weeks PMA.

### 4.2. Photoreceptor Layer Development [7, 16, 18]

The photoreceptor development is another aspect that has been better understood with the help of SD OCT, by providing a timeline of the development imaged in vivo. The height of the photoreceptor layer increases progressively from infancy to adulthood. This occurs rapidly after 38-week PMA in all the regions and especially in the cone-dense fovea. Furthermore, even if there is a delay in the inner retinal layer migration, this does not appear to affect or delay the development of the PRL complex.

Photoreceptor subelements are subcellular structures that can be imaged with the current available SD OCT devices. These structures include the external limiting membrane (ELM), the IS/OS (inner segment/outer segment) also known as the ellipsoid zone (EZ), and photoreceptor outer segments to RPE (OS-RPE) or interdigitation zone (IZ). The timelines when these layers “appear” or more correctly “can be imaged” varied considerably and are obviously a function of the resolution of the currently available devices. For example, in a study performed in the United States [16], the ELM was not observed until 42 weeks, whereas in a cohort of babies born with a heavier birth weight, it was imaged between 40-41 weeks [5].

**Figure 3:**
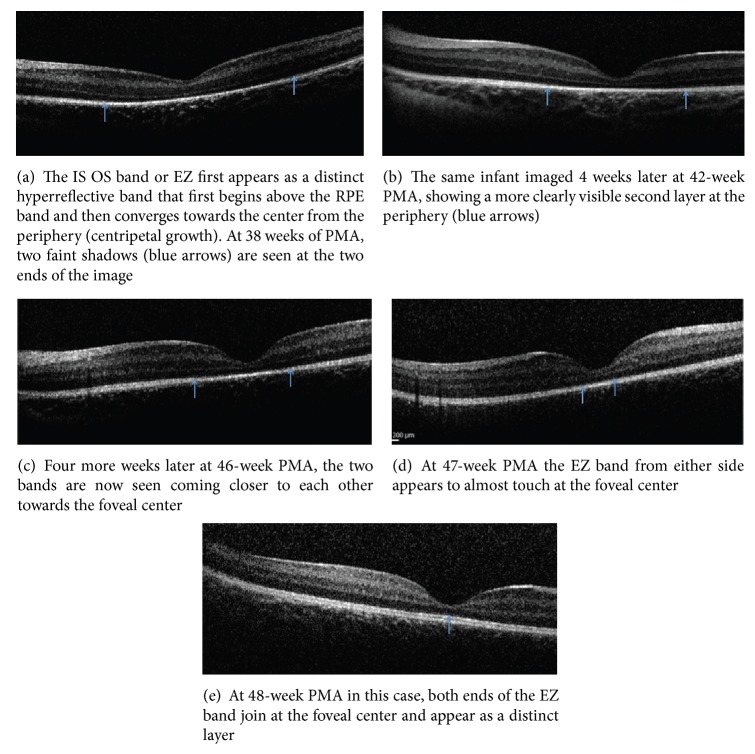
The centripetal growth of the IS-OS or EZ band from the periphery to the foveal center at different age groups imaged on the same infant (photo courtesy of Figures 3(a)–3(e) from Figures 90.2–90.6, Page 259–360, Chapter 90 of [59]).

In contrast to adult retinas, the photoreceptor layer in infants is initially thinner in the foveal center. As the infant grows, there is a progressive centripetal growth of the photoreceptor subcellular structures that extend into the foveal center. These layers include the IS-OS or EZ, the ELM, and the OS-RPE, respectively. The EZ band is a low reflective band barely elevated from the RPE outside the fovea imaged as early as 33 weeks PMA, which continuously thickens and moves towards the foveal center. The EZ is at the foveal center in 47% of term infants but in only 14% of preterm infants [18]. The process of central growth of the EZ band towards the foveal center at different ages in a premature infant is depicted in Figures 3(a)–3(e).

The RPE layer is seen as a hyperreflective layer after 31 weeks of PMA. The ELM has been variably noted in Asian Indian infants as early as 40.2 weeks and at 42 weeks in Caucasian infants [5]. However the time of imaging may vary with the imaging devices, the protocols, and software used in its capture. The OS-RPE or the IZ is the second subtle hyperreflective band between the IS/OS junction and the main RPE reflex. This is believed to be the interface between the photoreceptor outer segments and the RPE microvilli (OS/RPE) and differentiates at the apical side of the RPE much later in childhood. With these changes, the apical microvilli grow and change the interface with the photoreceptor outer segments. The band is not visible over the macula at term birth [18]. It is unclear at this time, about the exact age when this band matures into a layer, and is speculated to happen as late as the end of the first decade or in early adulthood.

**Figure 4:**
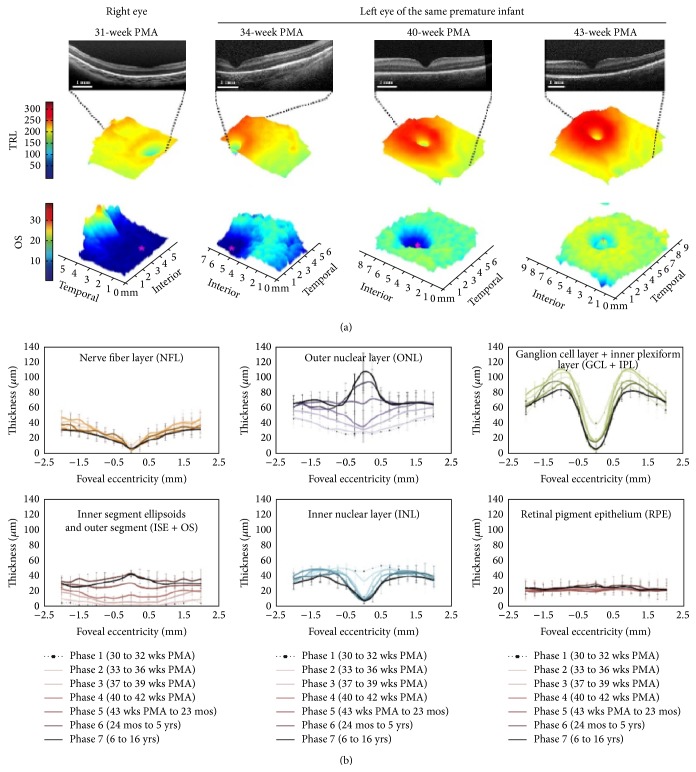
3D map of retinal layers and their dynamic changes with age in a neonate. The lower portion of the image has a segment on inner segment ellipsoid (photo courtesy: for the top of the figure (color maps), taken from Figure 2(a), Page 2320 of [16]; for the bottom portion of the figure (graphs), taken from Figure 2, Page 782. of [17]).

A map of retinal layer development in different ages and graphical depiction of foveal development is shown in Figure 4.

### 4.3. Histological Correlation of SD OCT Findings

Correlation and interpretation of neonatal SD OCT findings have come from histopathology and immunolabeling of postmortem eyes of neonates and children. [19]. Hendrickson et al. showed that at 22 weeks of fetal gestation, the fovea is a five-layered region with a thick GCL and a thin outer nuclear layer. After 25 weeks, the foveal pit begins to develop and invaginates into the INLs. Between fetal weeks 28–37, there is further deepening of the foveal pit, which thins the GCL, IPL, and INL compared to the layers surrounding the pit. Postnatally, the foveal pit is wider and shallower than before birth and has displaced most of the inner layers. The inner segment is longer and narrower than before birth and there is a short outer segment.

Over the postnatal period in the first 1 year of life, the pit becomes wide and shallow with no neurons in the center except for cone cell bodies. The OPL grows in size due to the increased number and length of the cone axons. There is more cone packing in the center as well. The final maturation continues through childhood. The foveal center has thin and long cones and the rod outer segments too become long with eccentricity.

Vajzovic et al. [17] compared the morphology with the layer thickness of the retinal SD OCT validating it as a reliable tool to assess foveal development and disease. The salient features pertinent to the foveal development that were reported are as follow: (1) there is progressive increase in the neurosensory retinal thickness from 30 weeks of postmenstrual age to 16 years and (2) preterms demonstrate shallower foveal pit and short, underdeveloped foveal photoreceptors.

Studies correlating histopathological structure with SD OCT [20–23] help us better understand in vivo anatomy. The foveal pit is the most characteristic feature of the human fovea and is caused by lateral displacement of the IRLs. During this process, the cones are tightly packed and elongate, migrating centripetally to the foveal center. The foveal avascular zone (FAZ) emerges by the 25th week PMA and the pit is also defined around this time. Further during the gestational development, there is inner retinal migration and reduction in the number of ganglion and bipolar cells at the foveal center. In the final trimester of pregnancy, the photoreceptors are more mature in the parafoveal and perifoveal area compared to the foveal center and the RPE interdigitates during this time with the outer segment as well. After birth, the foveal pit continues to get remodeled and is believed to reach maturity by 18 months of age. However, photoreceptors continue to elongate in the fovea postnatally with greater maturity in term born infants than very preterm infants imaged at term equivalent age [18]. The photoreceptors are said to reach the lower range of adults by four years of age.

Dubis et al. [24] showed the mapping of normal foveal development using SD OCT and histologic examination. The photoreceptor and foveal pit maturation showed variability even in age-matched individuals. Even through the 43rd week PMA, the IRLs persisted although the pit deepened and widened. In their series, the youngest infant with a fused inner retina with complete excavation of all inner retinal layers was 52nd week PMA or the third corrected month (assuming 40 weeks as term gestation). The outer retina in their series resembled adult-like bands on the SD OCT at 17 months of age.

Vajzovic et al. recently compared the photoreceptor development on SD OCT of preterm infants born with a gestational age of <32 weeks with that of term infants. Preterm infants between the PMAs of 37–42 weeks were imaged. The EZ developed in the foveal center only in 14% of preterm babies compared to 47% of the term babies. This was fewer in infants with macular edema than those without. For those with incomplete EZ development, the distance from the foveal center was less in term infants (mean 492 microns) compared to preterm infants (mean 783 microns). The cone outer segment tips (COST) layer was not seen in any infant in the age group studied. The study highlights the fact that photoreceptor inner and outer segment development is delayed in preterm infants compared to term infants and may actually explain the differences in visual function in them [18].

## 5. Spectral Domain Optical Coherence Tomography in Disease

As majority of the reports thus far pertain to the study of SD OCT in retinopathy of prematurity, this will be discussed in detail and then other non-ROP conditions will be summarized.

### 5.1. SD OCT in Retinopathy of Prematurity

**Figure 5:**
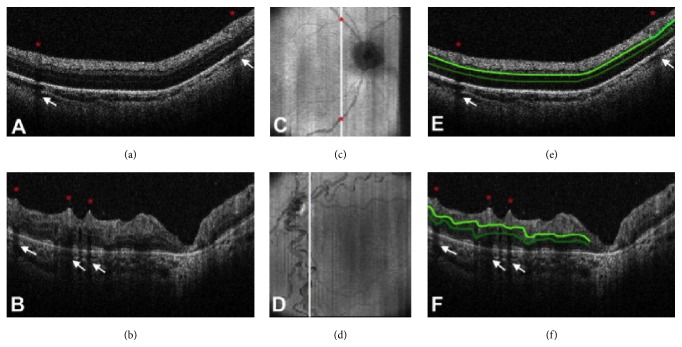
The spectral domain optical coherence tomography (SD-OCT) scans from a 31-week postmenstrual age (PMA) neonate (a) with retinopathy of prematurity (ROP) zone II, stage 2, and normal vasculature per clinical examination and a 48-week PMA neonate (b) with ROP zone II, stage 3, and plus disease. No vessel elevation is seen in (a) and severe vessel elevation is seen in (b). Retinal images (c, d) created from axial compression of SD-OCT scans. (e) and (f) contain the same scans as (a) and (b), respectively, but highlight the smooth retinal layer contour in (e) and the scalloped pattern on (f).* Red asterisks* are placed over vessels, and the corresponding location on the retinal image is shown on (c) and (d).* White arrows* point to shadow produced by the corresponding vessels. ((e) and (f)) The top* light green line* represents the inner plexiform layer, and the* bottom dark green line* represents the outer plexiform layer. (Photo courtesy Figure 2, Page 129, of [32].)

The gold standard for ROP screening and management has been the examination of infants with the indirect ophthalmoscope. However, fundus imaging using wide-field retinal cameras has offered several advantages including providing telemedicine and remote screening of these infants as well as for medicolegal documentation, training, and education [25–28]. More recently, SD OCT imaging has been reported to demonstrate clinically unseen or poorly detected retinal features [3, 6, 10], which include the following:

1. Preretinal structures are useful in detecting neovascularization (NV) or posterior NV which often accompany the more aggressive forms of zone 1 disease or aggressive posterior ROP (APROP) [3, 6].
2. Clinically undetected structures that have been imaged on SD OCT include retinoschisis [29], vitreoretinal interface and epiretinal membranes [30], retinal detachment [6], retinal pigment epithelium changes, and atrophy [30].
3. OCT has also been used to differentiate and prognosticate macular involvement in advanced cases of ROP, which can potentially change the diagnosis from stage 4A to 4B [2].
4. Postsurgery, after lens sparing vitrectomy, SD OCT has been used to monitor the reattachment of the macula, enabling better prognostication and patient education and allowing the correlation between structural and visual improvement [31].
5. OCT guided laser photoablation of residual neovascular fronds that are difficult to detect on clinical exam after the initial laser in extensive cases of APROP has been reported in Asian Indian infants [4].
6. In an attempt to correlate SD OCT features of ROP with vascular changes documented on fundus images, Maldonado et al. [32] have investigated the role of 3D-volume analysis and proposed the vascular abnormality score by OCT (VASO). The score is based on the presence of at least one of the following features on SD OCT: (a) retinal vessel elevation, (b) scalloped retinal layers, (c) hyporeflective vessels, and (d) retinal spaces. The score was noted to be higher in eyes with plus disease than those without. These infants had a greater retinal surface elevation, which matched with en face retinal vascular patterns giving us the evidence that vascular dilatation and tortuosity effect perivascular tissue (Figure 5).

### 5.2. SD OCT and Macular Edema

Macular edema or foveal edema has been the subject of recent research since it was incidentally detected in clinically normal looking foveae. Owing to the current lack of evidence of the exact etiology, extent, course, and effect of this entity, there is considerable scope for more research on this subject.

Vinekar et al. [5, 7] detected what they described as “foveal disruptive” changes on the SD OCT in clinically normal looking foveae in a cohort of Asian Indian premature infants undergoing ROP screening. These changes were detected on a modified hand-held version of a standard, adult, tabletop OCT device, Spectralis (Heidelberg, Germany). Of the 79 eyes included in the study with clinical normal foveae and Stage 2 ROP, 23 (29.1%) eyes appeared to have abnormal foveal changes characterized as “Pattern A” which involved a dome shaped foveal elevation and cystoid spaces with highly reflective intervening vertical septae observed in 12 (52.2%) eyes. “Pattern B,” which was characterized by preservation of the foveal depression with fewer intraretinal cystoid spaces was seen in 11 (47.8%). “Abnormal foveal changes” were observed in infants with Stage 2 ROP and not in Stage 1 ROP or non-ROP infants. These changes peaked at 37 PMA and all self-resolved without therapy or intervention by the 52nd week PMA or the third corrected month. Two etiologies were proposed, one of increased vascular endothelial growth factor (VEGF) and the other a mechanical factor. Ethnic variability and the clinical relevance are still under investigation.

Similar “macular edema like” changes have been reported in the Caucasian and mixed ethnic cohorts in North America [10–12]. Lee et al. [30] reported cystoid changes in the inner nuclear layer of 39% of the OCT sessions in their study of 76 eyes. None of these changes were detected clinically on routine indirect ophthalmoscopy. The difference in macular edema in the Asian Indian [7, 31] and the Caucasian population [9, 16] is summarized in Table 2.

Maldonado et al. in 2012 [33] studied the association of severity of cystoid macular edema (CME) with ROP and other systemic health factors unrelated to ROP. They too hypothesized that edema could be a marker of elevated active intravitreal VEGF. Forty-two infants were assessed for the severity of CME. The measures of severity included CFT, retinal layer thickness and foveal-to-parafoveal ratio. These parameters were correlated with the stage of ROP, plus disease, and treatment status as well as systemic factors such as Apgar score, surgery for patent ductus arteriosus, culture-proven sepsis, surgery for necrotizing enterocolitis, and the presence of intraventricular hemorrhage, periventricular leukomalacia, bronchopulmonary dysplasia, or hydrocephalus. They found that 50% of these infants had edema irrespective of the severity of ROP or the systemic factors under consideration.

Extending the finding of these edemas in more advanced stages of ROP, Dubis et al. [34] in a prospective, observational case series evaluated subclinical macular findings in premature infants at risk of ROP using the hand held system. This study demonstrated the presence of CME in 25 of the 49 infants (51%) imaged in the neonatal intensive care unit. The edema was found in Stages 0, 1, 2, 3, and 4A of ROP thereby concluding that the stage of the disease may not be associated with macular edema thereby concurring with the findings of Maldonado et al. [33].

More recently, Rothman et al. [35] evaluated the association of CME and neurodevelopmental outcomes in very preterm infants at 18 to 24 months corrected age. The imaging was done during routine ROP screening and the Bayley scores for neuro- and cognitive development were assessed at 18 to 24 months. Of the 53 children evaluated with Bayley scores, 31 children who had CME as infants had a lower mean score on cognitive, motor, and language subscale when compared to the children who did not have edema as infants. This paper throws light on the association of cystoid macular edema and neurodevelopment and the possible potential for retinal examination of macular edema as an indicator of neurodevelopmental health in infants.

Erol et al. [36] in a study from Turkey reported macular edema in 139 eyes of 190 premature infants imaged. Of their study cohort 126 eyes had ROP. They noted that 54% of eyes with ROP had edema compared to 31% without ROP. With increasing stage of ROP, there was a higher incidence of macular edema, that is, 46.3% in stage 1, 57.1% in stage 2, and 87.5% in stage 3 ROP.

Recently, Vinekar et al. [31] reported the visual and refractive outcome of infants with macular edema by following their initial cohort of infants with edema and comparing them with age matched premature infants with ROP (positive control) and without ROP (negative controls). They found that the visual acuity was lower in infants with macular edema compared to the other two control groups throughout the study period of one year, but statistically significant only at 3 months. The edema cohort was more hyperopic compared to the other two groups between 3 and 12 months of age and was possibly due to visual disturbances caused at a critical time of foveal development. On the other hand, in a small series including infants with and without edema, Rothman et al. [9] correlated the posterior segment microanatomy from perinatal SD OCT to visual acuity, brain abnormalities, and neurodevelopment. Those without edema had better vision compared to those with edema. They also found sensorimotor deficits and neurodevelopment changes in the group with edema. In one infant in the study the edema persisted through nine months.

### 5.3. Shaken Baby Syndrome (SBS) [10, 37–39]

SD OCT provides valuable information in SBS. Vitreoretinal membranes seen on imaging support the direct mechanical effect as one of the main pathophysiological theories. Preretinal blood, localized vitreous detachment, premacular folds, and attachment of the vitreous to the ILM at the apices of the perimacular folds are features that are detected. The presence of perimacular folds and hemorrhagic macular retinoschisis has been linked to poor visual outcome [10].

### 5.4. SD OCT in Other Retinal Conditions in Infancy

Other retinal conditions in infants and young children that have been reported are combined hamartoma of the retina and RPE [40–45], incontinentia pigmenti, retinal dystrophies and degenerations [46–55], retinoschisis, and other syndromes [56]. Systemic conditions like liver failure and neonatal hemochromatosis [57], causing macular changes, suggest that OCT can image retinal manifestations of ocular and systemic disease even when it is not clinically discernible [58].

## 6. SD OCT and the Choroid

SD OCT studies of the choroid have been extensive in adults, especially with the use of techniques such as enhanced depth imaging (EDI) that provides better visualization of the layers. However, there have been only a handful of studies published, which study the choroid in infants and children [60, 61]. Park and Oh analyzed the choroidal thickness profiles of children between the ages of 4 and 10 years subdividing them into preterm and full-term children [60]. From the 31 preterm and 30 full-term born children it was found that those that belonged to the preterm group had decreased choroidal thickness 3 mm temporal to the fovea.

More recently, Moreno et al. reported the feasibility of choroidal imaging without the use of EDI in both preterm and term infants and concluded that the presence of melanin in the RPE and less developed pigmentation in the choroid at that early age does not hinder choroidal imaging in preterm infants without an advanced stage of ROP. It was also noted that the choroidal thickness increased with age. However preterm infants had a thinner choroid when compared to term infants at the same age and adults [61].

## 7. SD OCT and the Optic Nerve

Our knowledge and understanding of the optic nerve and its development so far come from various histological studies [62]. With the advent of the portable hand-held SD OCT we can now perform imaging at the clinic, providing us with real-time information of the optic nerve. This technology has led to a number of studies of the optic nerve in adults; however the application of SD OCT to study the infant optic nerve is still limited.

OCT studies have looked at the development of the optic nerve and its association with possible demographic factors. The study by Allingham et al. [63] looked at the variation in the optic nerve parameters in full-term infants of different races and found a significant difference in the cup-disc ratio at the time of birth between white, black, and Hispanic babies which could be a normal trend for each of the races, thereby defining a normative range among the newborns.

Another study by Tong et al. [64] described the optic nerve parameters in term and preterm infants and their association with neurodevelopment and prematurity. The study showed that preterm infants with a risk of developing ROP had a larger cup-disc ratio when compared to term infants. In another study, the average retinal nerve fiber layer (RNFL) thicknesses in healthy, full-term neonates has been assessed and found to be normally distributed at 1.5 mm radial distance from the optic nerve. Interestingly, they found no significant difference of RNFL thickness with respect to race, gender, gestational age, or birth weight [65].

## 8. SD OCT and Visual Acuity Correlation in Infants

Although infant SD OCT imaging helps us better understand the anatomy, subclinical pathology and foveal development in normals and in disease, there is lack of evidence that correlates visual acuity with these retinal structures demonstrated on OCT. A retrospective analysis of 62 (54.4%) premature infants who had their visual acuities correlated with foveal layers at different time intervals in the first year of life showed that visual acuity correlated positively with IRL fusion, presence of the ELM, and the outer segment layer [66].

In another prospective study, 50 Asian Indian premature infants with and without ROP were followed through their first year of life and imaged at regular intervals of 3 months. The group without ROP showed better visual acuity correlation with foveal layer development compared to those without foveal layer maturation. In the group with ROP, there was no significant difference between those with and without these layers [67]. Future multicenter and multiethnic studies that evaluate the influence of foveal development and visual acuity with and without disease are required.

## 9. Future Trends

With the expanding knowledge base on spectral domain optical coherence tomography in the practice of pediatric retinal disease, future trends are likely to include custom software to create three-dimensional data sets to map structures, shapes, and abnormalities which would provide spatial information for understanding normal anatomy, development, and pathologic events [68].

Newer imaging tools such as the OCT-angiography, adaptive optics, and oximetry are likely to be modified for the use in pediatric retina, much like the evolution of the hand-held OCT now in vogue for infants in the clinic setting. Intraoperative use of microscope integrated OCT devices [69] is likely to provide a surgical assist for better demonstration of the pathology during and in the immediate postoperative period. OCT is a step beyond the routine fundus imaging and has allowed us to see the unseen and learn the unknown. Further evolution of the technology is most certainly going to help us understand and manage our patients better.

## Figures and Tables

**Table 1:** Reference table for refractive error, axial length, change in reference arm position, and the relative length to an adult scan.

Group age	Refractive error (D)	Axial length (mm)	Increase in reference arm Δ Gen 3 engine (Readout units)^‡^	Increase in reference arm Δ/2 Gen 3+ engine (Readout units)^*¥*^	Relative scan length to adult scan length (%)
30–35 wks	−1	15.1	97	−48	63
35–39 wks	0.3	16.1	86	−43	67
39–41 wks	0.4	16.8	79	−39	70
0-1 mos	0.9	17.4	72	−36	73
1-2 mos	0.3	18.6	59	−29	78
2–6 mos	0.5	18.9	56	−28	79
6–12 mos	0.6	19.2	52	−26	80
12–18 mos	0.7	20.1	43	−21	84
18 mos–2 yrs	0.9	21.3	30	−15	89

^‡^With respect to Gen 3 Engine (Older Bioptigen system), a higher number is equal to a shorter reference arm length on each system used.

^*¥*^The per turn change in reference path length is two times that of the Gen 3 reference arm. The direction is reversed so that larger numbers refer to larger distances.

**Table 2:** Comparison of macular edema in premature infants reported by two groups in the United States of America and India, respectively.

	Maldonado et al. [16]^*∗*^ Rothman et al. [9]^*∗∗*^	Vinekar et al. [7] Vinekar et al. [31]^*∗∗∗*^
Number of patients	42	74
Birth weight (mean)	760 grams	1282 grams
Gestational age (mean)	26 weeks	31 weeks
Race	Mixed race (African American 52%, White 40%, and Hispanic 7%)	Asian Indian (100%)
ROP stages included	0, 1, 2 and 3	0, 1 and 2
Incidence of macular edema, *n* (%)	21 (50)	12 (16) (23 eyes of 146 eyes with ROP)
CME characteristics	Bulging fovea 13 (62)	Pattern A 52% Pattern B 48%
Central foveal thickness	166 (91–499) microns	206 (108–304) microns
CME resolution (latest weeks)	43^*∗*^ 9 months^*∗∗*^	52
Visual acuity correlation (earliest checked)	9 months^*∗∗*^	3 months^*∗∗∗*^
Age range of visual correlation	9 months–5 years, (retrospective)	3–12 months (prospective)
Visual acuity mean at 9 months in logMAR	With edema (2): OD = 1.3, 1.3 OS = 1.9, 1.3 Without edema (2): OD = 0.8, 1 OS = 1, 1	Edema + ROP (11) = 1.30 Without edema + ROP (16) = 1.20 Preterm (17) = 0.98

## Abbreviations

APROP: Aggressive posterior retinopathy of prematurity
CCD: Charge coupled device
CFT: Central foveal thickness
CME: Cystoid macular edema
COST: Cone outer segment tips
EDI: Enhanced depth imaging
ELM: External limiting membrane
EZ: Ellipsoid zone
FAZ: Foveal avascular zone
GCL: Ganglion cell layer
ILM: Inner limiting membrane
INL: Inner nuclear layer
IPL: Inner plexiform layer
IRL: Inner retinal layers
IS-OS: Inner segment/outer segment
IZ: Interdigitation zone
NV: Neovascularization
OPL: Outer plexiform layer
ORL: Outer retinal layers
OS-RPE: Photoreceptor outer segments to RPE
PMA: Postmenstrual age
PRL: Photoreceptor layer
RNFL: Retinal nerve fiber layer
ROP: Retinopathy of prematurity
RPE: Retinal pigment epithelium
SD OCT: Spectral domain optical coherence tomography
VEGF: Vascular endothelial growth factor.
